# The Role of the Dopamine Melanin Pathway in the Ontogeny of Elytral Melanization in *Harmonia axyridis*

**DOI:** 10.3389/fphys.2019.01066

**Published:** 2019-08-27

**Authors:** Xu Chen, Da Xiao, Xiaoyan Du, Xiaojun Guo, Fan Zhang, Nicolas Desneux, Liansheng Zang, Su Wang

**Affiliations:** ^1^Jilin Engineering Research Center of Resource Insects Industrialization, Institute of Biological Control, Jilin Agricultural University, Changchun, China; ^2^Institute of Plant and Environment Protection, Beijing Academy of Agricultural and Forestry Sciences, Beijing, China; ^3^French National Institute for Agricultural Research, University of Côte d’Azur, Sophia Antipolis, France

**Keywords:** DOPA decarboxylase, melanin, elytra, fecundity, *Harmonia axyridis*

## Abstract

Polymorphic melanism in insects is a conspicuous phenotype which is derived from specific genotypes, and might be central to speciation processes via assortative sexual selection. At the molecular level, melanism in insects is attributed to the melanin pathway. DOPA decarboxylase (DDC) protein encoded by the *DDC* gene plays a central role in dopamine-melanin synthesis, the main component of melanin in insects. Although the mechanism of melanism has been elucidated in holometabolous insects, other physiological processes coupled with melanin synthesis are unknown. Herein, we identified *DDC* from the Asian multi-colored ladybird (*Harmonia axyridis*), an ideal holometabolous insect for studies of melanization due to highly variable color on their elytra. Analyses revealed that *HaDDC* (the *DDC* gene of *H. axyridis*) was constitutively expressed throughout all developmental stages. We performed RNAi technique to examine the melanin synthesis pathway of elytra in *H. axyridis*. The transcript levels of *HaDDC* were significantly suppressed after the injection of double-strand RNA of *HaDDC* (ds*HaDDC*) at 300 ng/individual in third instar larvae. Silencing *HaDDC* in third instar larvae did not result in mortality nor significantly affect pupation and eclosion. We further demonstrated that all adults of *H. axyridis* (forms *succinea*, *spectabilis*, and *conspicua*) with *HaDDC* silenced in third larvae showed abnormal phenotype which emerged as decreased elytra melanin. However, melanin was still observed in other parts of the adults such as head or pronotum. These results demonstrate for the first time that dopamine-derived melanin is the main contributor in elytra melanization in *H. axyridis*. Additionally, we provide evidence for *DDC* in regulating fecundity by showing that silencing of *HaDDC* in third instar larvae significantly reduced female egg-laying and egg hatching. As such, *DDC* is likely pleiotropic in respect of its role in melanin production and fecundity processes. These findings bring novel insights into melanin production in holometabolous insects, and contribute to the framework on which further studies may be conducted on the mechanism of pigment production and patterning in various types of insect coloration.

## Introduction

Pigmentation is a conspicuous and highly variable feature of insect physiology with important implications for a variety of behavioral, physiological, and reproductive performance which would be involved in evolutionary processes (Wigglesworth, 1954; Nijhout, 1991; Vershinin, 1999; Liu et al., 2014). Melanin is a ubiquitous pigment in the animal kingdom, functioning in the formation of adaptive color patterns (Liu et al., 2015), protection against ultraviolet radiation, and even immune responses to pathogens and parasitoids (Dubovskiy et al., 2013). For example, many insects employ melanin in the encapsulation of foreign objects, bacteria, nematodes, or parasitoids within their hemocoel (Dimopoulos et al., 2002; Anderson, 2010). Thus, the most established mechanisms about pigmentation may be concerned around melanism.

Melanin is the component of most black spots on the insect body (Hackman, 1967; Kumar and Hopkins, 1987), and synthesis process of melanin has been well characterized in insects (Koch et al., 2000; Wittkopp et al., 2002; Futahashi et al., 2008; Arakane et al., 2009). The melanin synthesis is a complex biochemical process involving a series of enzyme reactions. Firstly, the tyrosine converts to DOPA (dihydroxyphenylalanine) by tyrosine hydroxylase (TH), then it used to produce DOPA melanin. Alternatively, DOPA can be further converted to dopamine by DOPA decarboxylase (DDC). Finally, phenol oxidases (POs) covert dopamine into its quinones, then which are converted to dopamine melanin (Wright, 1987; Lemonds et al., 2016). DDC, a pyridoxal-5-phosphate-dependent enzyme, catalyzes the conversion of DOPA to dopamine which is an important neuro-transmitter (Eveleth et al., 1986; Scholnick et al., 1986). This enzyme has been studied extensively in most organism, especially in insect, DDC is primarily expressed in epidermal, neural and ovarian cells of *Drosophila* (Beall and Hirsh, 1987; Konrad and Marsh, 1987; Vermeulen et al., 2006), and it is involved in embryonic development, cuticular sclerotization, molting and chorion tanning in *Manduca sexta* (Hori et al., 1984; Hiruma et al., 1985; Hiruma and Riddiford, 1993; Li and Christensen, 1993). Previous studies have showed that DDC is a key enzyme regulating ecdysone which play important role in the process of molting of *Bombyx mori* (Wang et al., 2013). It seems that the DDC plays a junctive role between the melanin biosynthesis and other key phenotypes or essential developmental/physiological performances, which could be related to evolutionary characteristics.

The Asian multi-colored (Harlequin) ladybird, *Harmonia axyridis* (Coleoptera: Coccinellidae), has been employed in field and greenhouse crops for biological control of insect pests since the early twentieth century (Katsanis et al., 2013; Xiao et al., 2017). Although this predator has become a component of integrated pest management (IPM) of many pests in its native region, however, it has also become an aggressive invasive species in the introduced regions (Koch, 2003; Majerus et al., 2006; Soares et al., 2008; Wang et al., 2009; Roy et al., 2016). Similar with most holometabolous insects, there is no variation in phenotype in the larval and simple thermal melanism in the pupal stage in *H. axyridis*, however, adult *H. axyridis* display highly diverse elytral color morphs formed largely by different patterns of melanin deposition, making the species a useful model for investigating melanin synthesis and deposition in insect elytra (Kuwayama et al., 2006; Ando and Niimi, 2019). In addition, there is significant difference of phenotype of *H. axyridis* in different seasons, moreover, assortative mating based on melanic and succinic phenotype was found in *H. axyridis* population (Wang et al., 2015).

Thus, the *H. axyridis* was considered as excellent model of phenotype evolution for discuss the relationship between high polymorphic diversity and specifically physiological/biological characteristics. Remarkably, recent genome studies showed that a single GATA transcription factor gene *pannier* can regulate pigmentation patterns during elytral development in *H. axyridis* (Ando et al., 2018; Gautier et al., 2018). However, which melanin is the component in the elytra of *H. axyridis* and if the melanin formation may be linked with any metabolism processes still unclear. In this study, we took the advantage of *H. axyridis* for its robust RNAi to examine the effect of *HaDDC* silenced on elytra melanization and other related physiology process in *H. axyridis*. Our studies provided strong evidence that dopamine-melanin is the major melanin component in the elytra and *HaDDC* has potential function in regulating fecundity in *H. axyridis*.

## Materials and Methods

### Insect Culture

*Harmonia axyridis* were derived from cotton fields (39°95′ N, 116°28′ E) on the experimental campus of the Beijing Academy of Agriculture and Forestry Sciences (BAAFS), Beijing, China, in May, 2013. Then we maintained inbreeding culturing in the lab and gained a colony with stable genetic morphs. The beetles were transported to a rearing room in the Applied Entomology Laboratory, Institute of Plant and Environment Protection, BAAFS that was climate-controlled using an automated environmental management system (Sunauto, Beijing, China). Here, they were reared in aluminum frame cages (50 cm × 50 cm × 50 cm) covered with 100-mesh plastic gauze at 25 ± 1°C, relative humidity 60%, and photoperiod 16 h:8 h (L:D). There are 40 pairs of adults in every cage, which were fed daily on cowpea aphids, *Aphis craccivora* Koch (Hemiptera: Aphididae) on leaves of seedlings of broad bean, *Vicia faba* L., cv. “LinCan-5” (Wang et al., 2013).

### Total RNA Isolation and Reverse Transcription

TRIzol reagent (Invitrogen, Carlsbad, CA, United States) was used for isolating total RNA from each sample. The RNA concentration was tested by using a NanoDrop 2000 spectrophotometer (Thermo Fisher Scientific, Waltham, MA, United States) at 260 nm. In order to remove possible genomic DNA contamination, the total RNA (1.0 μg) was treated with gDNA Eraser (Takara, Dalian, China), then first-strand cDNA was synthesized using the First Strand cDNA Synthesis Kit (Takara, Dalian, China) with oligo (dT)_18_ as the primer in a 20 μL reaction system. The first-strand cDNA was used for all subsequent analyses.

### Analysis of Expression Profiles by Reverse Transcription Quantitative PCR

The relative transcript levels of *H. axyridis* DDC (*HaDDC*) were analyzed by reverse transcription quantitative PCR (RT-qPCR) using SYBR Green with the Applied Biosystems^®^ Real-time PCR Instrument (ABI Laboratories, Hercules, CA, United States). In order to obtain the developmental expression profiling of *HaDDC*, samples were prepared from the embryos of 1–3-day old eggs, larvae of all four instars, and pupae ranging from 1 to 5 days in age. Total RNA was extracted from samples of each stage using TRIzol reagent (Invitrogen, Carlsbad, CA, United States), and 1.0 μg of total RNA was used in order to obtain the cDNA synthesis using the First Strand cDNA Synthesis Kit (Takara, Dalian, China). RT-qPCR primers were designed using the Primer premier 5 software based on *HaDDC* in NCBI (Accession number: KU820948), and the ribosomal protein S49 [*Harp49* (Accession number: AB552923)] in *H. axyridis* was used as an internal reference gene (Table 1).

**TABLE 1:** Primers used to synthesize dsRNA, and analyze transcript levels.

**Application of primers**		**Sequence (5′-3′)**	**Product length (bp)**
dsRNA synthesis	*dsGFP*(T7)-F	ggatcctaatacgactcactata gggTGACCACCCTGACCTAC	305
	*dsGFP*(T7)-R	ggatcctaatacgactcactata gggTTGATGCCGTTCTTCTGC	
	*dsHaDDC*(T7)-F	taatacgactcactataggg TATAAGGGAGAGGCGGGTTT	385
	*dsHaDDC*(T7)-R	taatacgactcactatagggGTA GCTTCACTCGCAGTCCC	
RT- qPCR	*HaDDC*(Q)-F	TAGTTGCCTTGCTTGGAG	141
	*HaDDC*(Q)-R	TTTGATTCGTCTGTGGGTA	
	*Harp49-F*	ACGGACTTCGGTAGGACG	130
	*Harp49*-R	CGCAGACAATCCCGAAA	

The optimized quantitative PCR program consisted of an initial denaturation at 95°C for 10 min, followed by 40 cycles of 95°C for 15 s, and 60°C for 1 min. After PCR, amplification specificity was verified by obtaining the dissociation curve, in which the samples were cooled to 55°C after denaturing, and melting curves obtained by increasing 0.5°C /10 s for each cycle with a total of 80 cycles until reaching 95°C to denature the double-stranded DNA. The specificity of each reaction was evaluated based on the melting temperatures of the PCR products. RT-qPCR was performed with three biological replicates, each with three technical replicates. The transcript levels of *HaDDC* were expressed as normalized transcript abundance using *Harp49* as an internal reference gene. The relative *HaDDC* transcript levels were calculated according to the 2^–△^^△^^*Ct*^ method (Xiao et al., 2014).

### Functional Analysis of *HaDDC*

RNA interference (RNAi) was performed to evaluate the role of *HaDDC* in *H. axyridis* development and elytral melanization. Double stranded RNAs (dsRNAs) were synthesized using MEGAscript^®^ RNAi Kit (Invitrogen, Carlsbad, CA, United States) according to the manufacturer’s instructions. Relevant information on the primers used for dsRNA synthesis is given in Table 1. Third instar larvae were selected for injections of *HaDDC* dsRNA (ds*HaDDC*) at doses of 300 ng/individual, similar numbers of the same life stages were injected with dsRNA of the green fluorescent protein gene (ds*GFP*) at the same dose to serve as controls. Mortality resulting from the injection of ds*GFP* was less than 10%. Each RNAi experiment was repeated with three biological replicates, each replicate with at least 40 insects. The injected insects were reared under standard conditions and phenotype was observed and recorded daily after injection. RT-qPCR was used to monitor the change in *HaDDC* transcript level after the injection. Four insects from each time point were then pooled as a sample for total RNA extraction. Each time point was analyzed with three biological samples and each sample was run with three technical replications.

To examine effect of *HaDDC* silenced on insect fecundity and gender-dependent effect of the RNAi, third instar larvae were injected with ds*HaDDC* or ds*GFP* as controls. After the injected larvae developed into adults, the individuals of the *spectabilis* were separated and paired as follow: (1) G – male + G – female: a male injected with dsGFP mating with a female injected with dsGFP; (2) D – male + D – female: a male injected with dsHaDDC mating with a female injected with dsHaDDC (300 ng/larva); (3) G – male + D – female: a male injected with dsGFP mating with a female injected with dsHaDDC; and (4) D – male + G – female: a male injected with dsHaDDC mating with a female injected with dsGFP (Figure 7A) (Lu et al., 2012). Each treatment consisted of 10 pairs of a male and a female, and each treatment was repeated three times. Eggs were collected for 20 days after mated 8 days post-eclosion, whereas egg hatchability was examined 3 days after eggs were collected.

### Image Processing

Microscopy images were taken using Zeiss Microscope SteREO Discovery V20 (Carl Zeiss, Germany), all using the same magnification, exposure time, and light intensity. Images were then selected for depiction of the most representative phenotypes.

### Statistical Analysis

The level of *HaDDC* transcript in RT-qPCR analysis was expressed as a percentage of the level in controls by dividing the relative expression value (REV) in the ds*HaDDC*-injected insects by REV in the ds*GFP*-injected insects and multiplying by 100. Percent data from developmental stages analysis, along with data from the RNAi experiments, were arcsine square-root transformed before being subjected to ANOVA followed by Tukey’s HSD test to separate means (ProStat software, Poly Software International, Pearl River, NY, United States).

## Results

### Developmental Stage Expression Profiles of *HaDDC*

**FIGURE 1:**
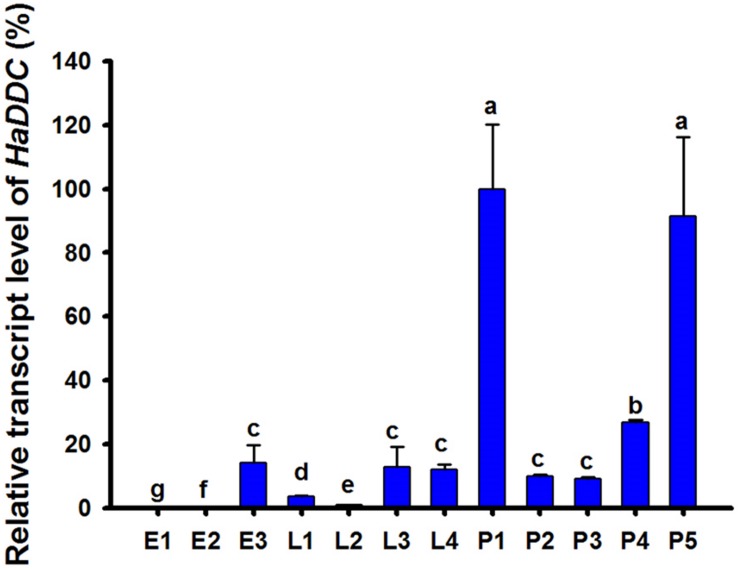
Relative transcript levels of *HaDDC* at different development stages of *H. axyridis* as determined by RT-qPCR. E1–E3 represent 1–3-day eggs; L1, L2, L3, and L4 represent first, second, third, and fourth instar larvae; P1–P5 represent 1–5-day pupae. *H. axyridis* ribosomal protein 49 gene (*Harp49*) was used as an internal reference gene to normalize the differences among the samples. Relative expression levels for *HaDDC* were calculated based on the highest expression of *HaDDC* in 1-day pupae (P1) as 100% in the development stage expression analyses. The results were presented as the mean and standard errors of three replicates (each was performed with a RNA sample prepared from four insects). Different letters above the standard error bars indicate significant differences based on ANOVA followed by Tukey’s HSD multiple comparison test (*P* < 0.05).

Analyses of stage-specific expression profiles of *HaDDC* using RT-qPCR in egg, larval and pupal stages revealed constitutive expression, with the highest average value of expression occurring on the 1st day of the pupal stage (Figure 1).

### RNAi of *HaDDC* in Third Instar Larvae and Its Effect on Survival

**FIGURE 2:**
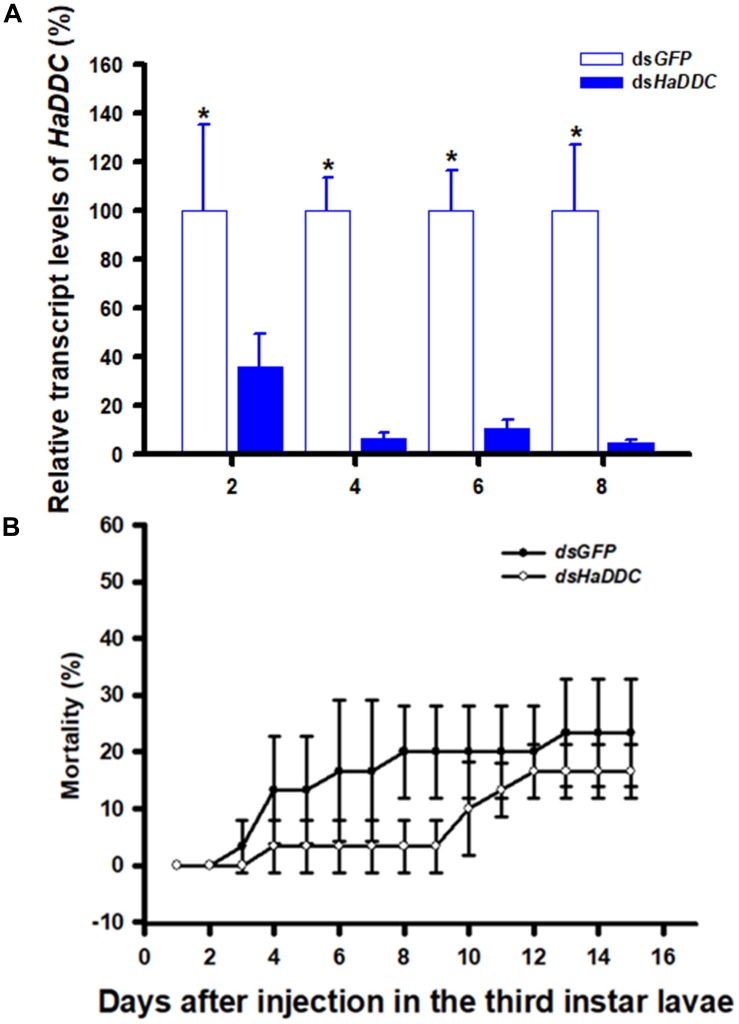
Time-dependent suppression of *HaDDC* transcript in third instar larvae of *H. axyridis* injected with ds*HaDDC* at 300 ng/larva or ds*GFP* at 300 ng/larva as determined by RT-qPCR **(A)**; and the time-dependent larval mortalities in the ds*HaDDC* and ds*GFP*-treated larvae **(B)**. The relative expression levels (%) are presented as the mean and standard errors of three replicates; each was performed with a RNA sample prepared from four insects and each sample was run with three technical replicates. The percent mortalities were also determined based three replicates; each replicate with at least 40 third instar larvae. Asterisk above the standard error bars indicate significant differences based on independent *t*-test (*p* < 0.05) within the same time point.

When third instar larvae were injected with ds*HaDDC* at 300 ng/individual, transcript levels of *HaDDC* were significantly reduced on 2, 4, 6, and 8 days after injection. The transcript levels of *HaDDC* were almost completely suppressed by day 4 after injection (Figure 2A). The injection of ds*HaDDC* in third instar larvae did not cause any significant mortality, even though the remaining *HaDDC* transcript level was only 4.69% of the control (Figure 2B).

### RNAi of *HaDDC* in Third Instar Larvae and Its Effect on Pupation and Eclosion

**FIGURE 3:**
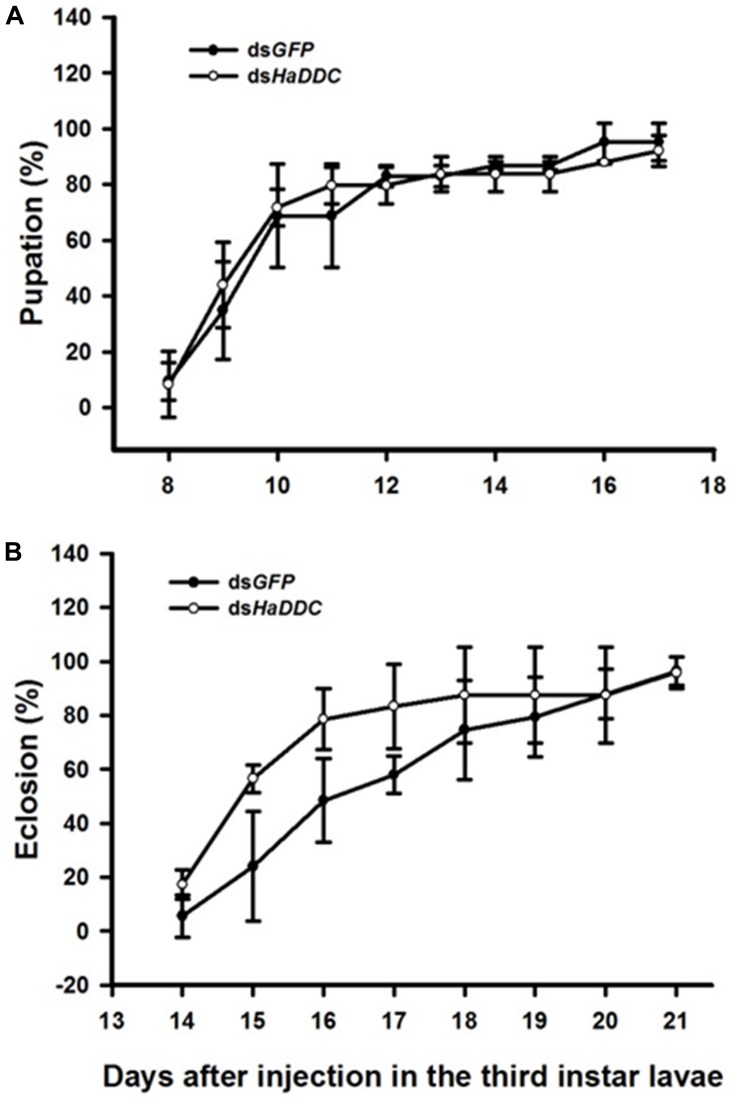
Time-dependent pupation rates **(A)** and eclosion rates **(B)** in the ds*HaDDC* and ds*GFP*-treated larvae. The percent pupation (or eclosion) rates were determined based three replicates; each replicate with at least 40 third instar larvae.

There was no difference in the percentage of third instar larvae that pupated successfully following injected with ds*HaDDC* when compared to control larvae injected with ds*GFP* (Figure 3A). Similarly, injection of third instar larvae with ds*HaDDC* had no significant effect on successful adult emergence compared to controls (Figure 3B).

### RNAi of *HaDDC* in Third Instar Larvae and Its Effect on Elytra Melanization

**FIGURE 4:**
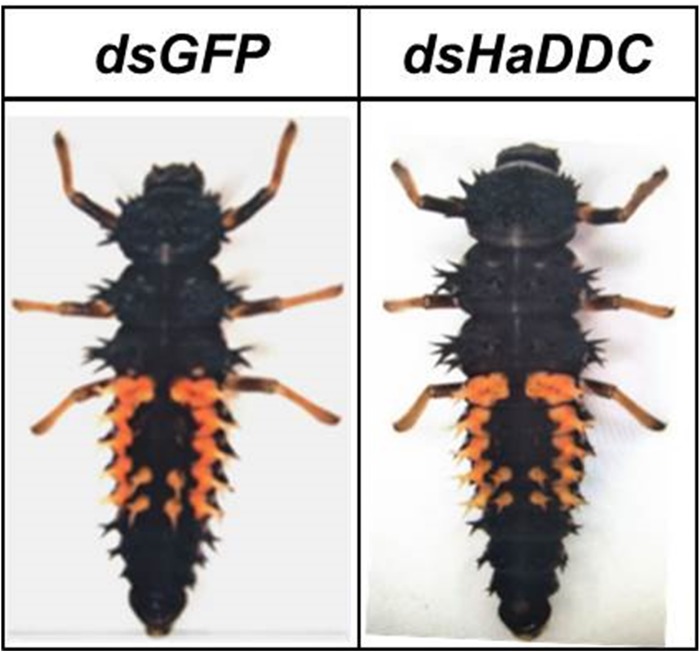
The fourth instar larvae phenotype of normal molting from the third instar larvae of *H. axyridis* injected with ds*HaDDC*.

**FIGURE 5:**
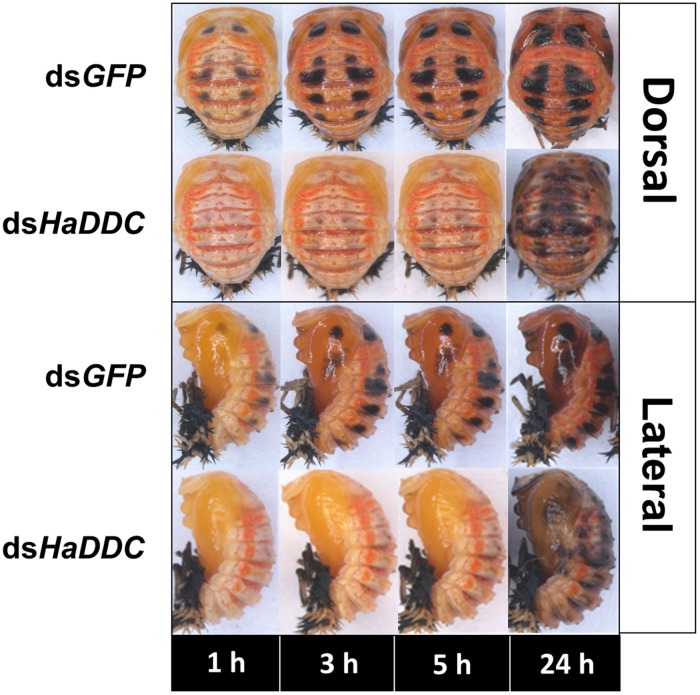
Dorsal and lateral views of pupae of *H. axyridis* that were injected with ds*HaDDC* in the third instar, compared to those injected with ds*GFP*.

**FIGURE 6:**
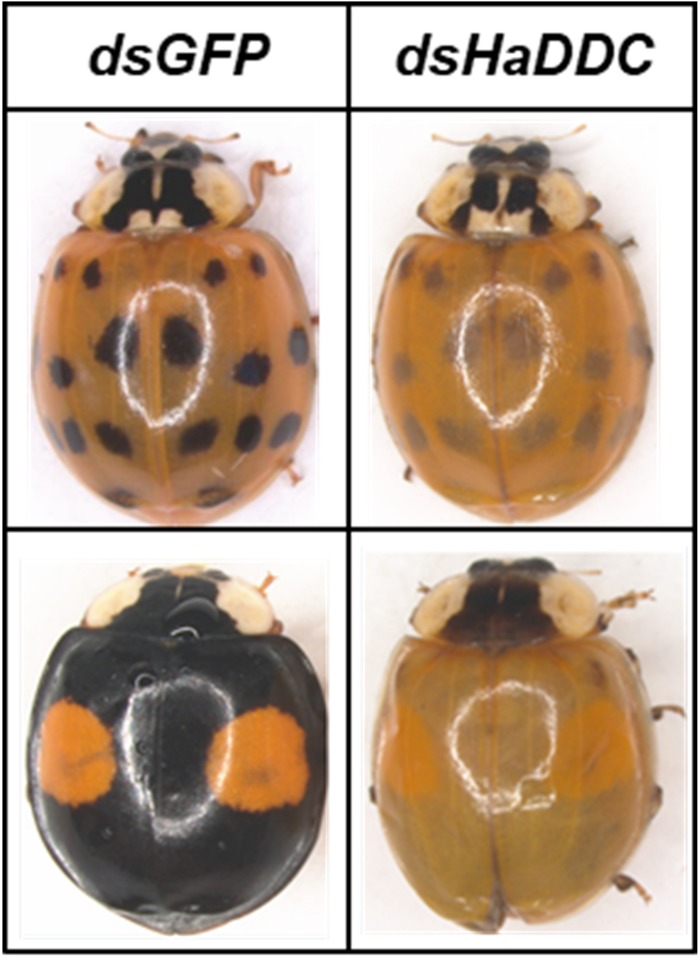
Adult *H. axyridis* that were injected with ds*HaDDC* in the third instar, compared to controls that were injected with ds*GFP* (form *succinea* above, form *conspicua* below).

When RNAi of *HaDDC* was performed in the third instar, the treatment larvae can normally molt into fourth instar and did not show any abnormal melanization when compared with controls that were injected with ds*GFP* (Figure 4). However, *HaDDC*-silenced pupae displayed a marked reduction in melanization compared to controls that became evident 0.5 h after pupation (Figure 5). In addition, emerging adults of all beetles showed reduced melanin of the elytra. However, melanin of the head and pronotum appeared normal (Figure 6).

### RNAi of *HaDDC* in Third Instar Larvae and Its Effect on Fecundity

**FIGURE 7:**
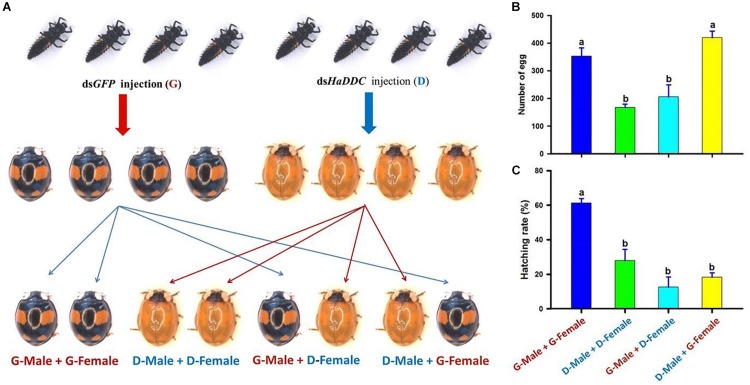
The gender-dependent effect of RNAi for *HaDDC* **(A)** on female egg laying for 20 days **(B)** and egg hatching **(C)** after the third instar larvae of *H. axyridis* (form *spectabilis*) were injected with ds*HaDDC*. G – male + G – female: a male injected with ds*GFP* mating with a female injected with ds*GFP*; D – male + D – female: a male injected with ds*HaDDC* mating with a female injected with ds*HaDDC* (300 ng/larva); G – male + D – female: a male injected with ds*GFP* mating with a female injected with ds*HaDDC*; D – male + G – female: a male injected with ds*HaDDC* mating with a female injected with ds*GFP*. The results are presented as the mean and standard errors of three replicates (each was performed with four pairs of the female and male). Different letters on the bars of the histogram indicate significant differences based on ANOVA followed by Tukey’s HSD multiple comparison test (*P* < 0.05).

We further investigated whether the injection of ds*HaDDC* in the third instar larvae would affect adult reproduction, and whether the RNAi effect would have transgenerational consequences. Although the injection of ds*HaDDC* in third instar larvae did not lead to the mortality of the ladybird, we observed dramatic decreases in the egg-laying (Figure 7B) and egg hatching (Figure 7C) when a female was from the larva injected with ds*HaDDC*. In contrast, such effects were not associated with any injections of ds*HaDDC* in the males. Thus, our observed effects of RNAi for *HaDDC* were clearly carried through the female rather than the male.

## Discussion

The mechanism of melanin biosynthesis has been well characterized in insects (Kramer et al., 1984; Futahashi et al., 2008; Arakane et al., 2009). DDC converts DOPA to dopamine, hence knocking down this enzyme should cause the irreversible loss of dopamine. In our study, we identified an *DDC* from *H. axyridis* (*HaDDC*) and used RNAi technique to investigate the role of DDC in cuticle melanin synthesis, survival and fecundity in *H. axyridis*.

After injecting ds*HaDDC* into the third instar larvae, the transcript levels of *HaDDC* were reduced by 99.55% (Figure 2A). *H. axyridis* was very sensitive to RNAi. In addition, our results are in agreement with previous reports that the transcript level and activity of DDC are significantly deduced in silenced mosquitoes (Huang et al., 2005). Beyond the production of melanin, DDC also has a predominant role in producing dopamine which is an important neurotransmitter. DDC-silenced *Armigeres subalbatus* showed abnormal phenotypes which are high mortality, abnormal movement and overfeeding (Huang et al., 2005). In *T. castaneum* study, injection of ds*TcDDC* into larvae produced a lethal pupal phenotype. When ds*TcDDC* was injected into young pupae, the resulting adults had abnormally dark brown body color, although with little mortality (Arakane et al., 2009). In our study, the injected amount of ds*HaDDC* was 300 ng/individual in third instar larvae, which resulted in no significant mortality, pupation rate and eclosion rate as compared with their respective controls, even though the *HaDDC* transcript level was reduced to only 4.69% of the control (Figures 3A,B).

Previous study reported that dopamine melanin is the major component of black pigments in *Oncopeltus fasciatus* (Liu et al., 2014). In *B. mori*, DDC played a key role in the synthesis of melanin and also involved in insect exoskeleton hardening (Noguchi et al., 2003). We performed the RNAi of dopa-decarboxylase (DDC) to examine whether the dopamine melanin synthesis is involved and to what degree it is generating the melanic dorsal coloration in *H. axyridis*. Silencing *HaDDC* in the third instar larvae clearly caused the loss of melanin in the elytra in *H. axyridis* (Figure 6). The above observation strongly suggests that the biosynthetic pathway of dopamine-melanin is related to the melanin of the elytra. Our study in combination with similar studies in *B. mori* (Wang et al., 2013), *T. castaneum* (Arakane et al., 2009), and *Periplaneta americana* (Lemonds et al., 2016), demonstrated that core components of the melanin pathway may be conserved among insect species (Sugumaran and Barek, 2016). In addition, the *DDC* silenced *H. axyridis* displayed normal melanic color in head and pronotum, thus we can speculate that elytra and other body wall use different melanin synthesis pathway during ontogenesis in *H. axyridis*.

New genome analysis indicated the expression of key gene *pannier* may strongly affect the melanin synthesis of *H. axyridis*, suppress the *pannier* gene in larval stage results in depletion of whole melanin in the adult body of *H. axyridis* (Ando et al., 2018; Gautier et al., 2018). However, our study showed different results that suppress the transcript level of *HaDDC* just result in depletion melanin of adult elytra in *H. axyridis*, and doesn’t affect the melanin synthesis in head and pronotum part (Figures 2A, 6). An enhanced understanding of the molecular genetics of melanism synthesis and deposition in insects could have far-reaching implications for insect physiology, given the broad array of pleiotropic effects that melanism can have on diverse traits of adaptive significance in evolutionary biology (True, 2003). Better understanding of the mechanism of melanin synthesis in insect will afford novel idea for the research relationship between phenotype formation and initiative selection.

The function of genes is always pleiotropic in tissues and life-history stages, so it is significative to study the molecular characteristics of genes related to pigmentation whether they have other physiological functions. DDC catalyzes the decarboxylation of dopa to dopamine. Dopamine is a catecholamine work as neurotransmitter that plays an important role in mating, fertility, circadian rhythms, endocrine secretion, aggression, learning and memory (Neckameyer, 1997; De Luca et al., 2003; Gruntenko and Yu Rauschenbach, 2008). In this study, when *HaDDC* was significantly suppressed in third instar larvae, we found significantly reduced egg-laying in adult (F_0_ generation) and egg hatching in F_1_ generation (Figures 7B,C). These results indicate that suppressing the transcript levels of *HaDDC* can significantly decrease fertility in *H. axyridis*. These results also support our conclusion that elytra melanin related genes have pleiotropy effects, additionally functioning in one of the critically important facets of insect development, and with a vital role in reproduction.

In summary, we uncovered the melanin synthesis pathway in the elytra of *H. axyridis*, one that showed strikingly different expression when compared to head and pronotum. Future comparative works should include exploration of the melanin synthesis pathway of head and pronotum in *H. axyridis*.

## Data Availability

All datasets generated for this study are included in the manuscript and/or the supplementary files.

## Author Contributions

DX, SW, and FZ conceived and designed the study. XC and XD performed the research data. XG performed the statistical analysis. DX and SW wrote the first draft of the manuscript. ND and LZ wrote sections of the manuscript. All the authors gave their final approval for publication.

## Conflict of Interest Statement

The authors declare that the research was conducted in the absence of any commercial or financial relationships that could be construed as a potential conflict of interest.
